# Diverse triggers, common outcome: Senescence in Fix⁻ *Medicago truncatula* nodules

**DOI:** 10.1093/plphys/kiaf518

**Published:** 2025-10-23

**Authors:** Alexandra Pál, Rui M Lima, Hilda Tiricz, Ferhan Ayaydin, Attila Kereszt, Éva Kondorosi, Edit Ábrahám

**Affiliations:** Microbial and Plant Genomics Research Unit, Institute of Plant Biology, HUN-REN Biological Research Centre, Szeged H-6726, Hungary; Doctoral School of Biology, University of Szeged, Szeged H-6726, Hungary; Microbial and Plant Genomics Research Unit, Institute of Plant Biology, HUN-REN Biological Research Centre, Szeged H-6726, Hungary; Microbial and Plant Genomics Research Unit, Institute of Plant Biology, HUN-REN Biological Research Centre, Szeged H-6726, Hungary; Agribiotechnology and Precision Breeding for Food Security National Laboratory, Institute of Plant Biology, HUN-REN Biological Research Centre, Szeged H-6726, Hungary; Microbial and Plant Genomics Research Unit, Institute of Plant Biology, HUN-REN Biological Research Centre, Szeged H-6726, Hungary; Microbial and Plant Genomics Research Unit, Institute of Plant Biology, HUN-REN Biological Research Centre, Szeged H-6726, Hungary; Synthetic and Systems Biology Unit, Institute of Biochemistry, HUN-REN Biological Research Centre, Szeged H-6726, Hungary

## Abstract

Nodule senescence in barrel medic (*Medicago truncatula*) can occur as a natural, developmentally regulated process or be triggered prematurely by environmental stress or ineffective symbiotic interactions. In this study, we examined five *M. truncatula* Fix⁻ mutants (*dnf4*, *dnf7-2*, TR183, TRV36, and TR36) that fail to fix nitrogen to determine whether they share common senescence-related traits. Our findings reveal that, despite distinct genetic defects, all mutants exhibit similar hallmarks of premature senescence: a rapid decline in the transcription of nitrogen-fixation-related genes (as indicated by *DINITROGENASE REDUCTASE* (*NifH)* expression), early degradation of bacteroids and symbiotic cells, recolonization of nodules by saprophytic rhizobia, premature closure of the nodule endodermis, impaired postmitotic differentiation of the symbiotic cells, and upregulation of senescence marker genes (*CYSTEINE PROTEASE 2* (*CP2)*, *CYSTEINE PROTEASE 6 (CP6)*, *CHITINASE 2*, and *PURPLE ACID PHOSPHATASE 22 (PAP22*). Neither symbiotic maintenance genes (*DEFECTIVE IN NITROGEN FIXATION 2* (*DNF2)*, *Symbiotic CYSTEINE-RICH RECEPTOR-LIKE KINASE (SymCRK)*, and *REGULATOR OF SYMBIOSOME DIFFERENTIATION (RSD*)) that inhibit plant defense responses nor the defense-related gene *PATHOGENESIS-RELATED PROTEIN 10.1 (PR10.1)* were upregulated, suggesting that premature senescence in these mutants is driven primarily by proteolytic activities rather than immune responses. These results indicate that early nodule senescence is a common feature of ineffective *M. truncatula–Sinorhizobium medicae* interactions, independent of the specific genetic mutation. Understanding nodule longevity and functionality may contribute to the development of strategies to enhance symbiotic efficiency in legumes for sustainable agriculture.

## Teaser

Diverse gene mutations leading to ineffective symbiosis in *Medicago truncatula* elicit premature nodule senescence as a general plant response.

## Introduction

Although nitrogen gas (N_2_) is a major component of the atmosphere, eukaryotic organisms cannot directly utilize it. Only diazotrophic bacteria and archaea possess the nitrogenase enzyme required to reduce atmospheric N_2_ into ammonia, a bioavailable nitrogen source. In response to nitrogen deficiency, legumes establish a facultative symbiotic relationship with nitrogen-fixing rhizobium bacteria, which provide the plant with reduced nitrogen compounds. This symbiosis begins with a molecular exchange between the plant and rhizobium in the rhizosphere, ultimately leading to bacterial infection and the initiation of nodule primordium formation (Geurts and Bisseling 2002).

Within the nodule cells, bacteria are released into the cytoplasm, enclosed by a plant-derived membrane known as the symbiosome or peribacteroid membrane, forming organelle-like structures termed symbiosomes (Oldroyd et al. 2011). As symbiotic cells mature, in species like *Medicago* forming indeterminate nodules, they undergo differentiation, resulting in a structured, zoned organization: the apical meristem (Zone I), infection zone (Zone II), interzone II-III (IZ), nitrogen-fixing zone (Zone III), and senescence zone (Zone IV) in older nodules (Vasse et al. 1990). During differentiation in Zone II and interzone II-III, symbiotic cells undergo repeated endoreduplication cycles, resulting in the formation of polyploid cells reaching an ∼80-fold increase in their final size to accommodate thousands of endosymbiotic bacteria (Cebolla et al. 1999; Vinardell et al. 2003). In barrel medic (*Medicago truncatula*) and related legumes, bacteroids undergo a terminal differentiation process characterized by genome amplification through endoreduplication, cellular enlargement, morphological changes, and a definitive loss of division capacity (Mergaert et al. 2006; Kondorosi et al. 2013). In *M. truncatula*, *Sinorhizobium meliloti* bacteroids exhibit a ∼24-fold increase in genome size, 5–10-fold cell enlargement, Y-shaped morphology, and enhanced membrane permeability (Mergaert et al. 2006). This process, regulated by the host plant, is synchronized with host cell development across multiple cell layers, ultimately leading to the fully functional nitrogen-fixing state in Zone III.

During developmental senescence in Zone IV, symbiosomes are progressively degraded and resorbed, followed by host cell decomposition. This process is accompanied by the proliferation of saprophytic bacteria released from degraded infection threads and the activation of stress- and defense-related genes (Van de Velde et al. 2006).

Beyond natural aging, nodule senescence can be triggered by various environmental stress factors, including water deficit (Gogorcena et al. 1995; González et al. 1998), high nitrate levels (Lorenzo et al. 1990; Swaraj et al. 1993; Escuredo et al. 1996; Matamoros et al. 1999), prolonged darkness (Gogorcena et al. 1997; Matamoros et al. 1999; Pérez Guerra et al. 2010), and exposure to phosphinothricin (Seabra et al. 2012). When wild-type Fix*⁺* nodules were wounded, intracellular rhizobia perished, and both defense and senescence marker genes were upregulated, indicating that these processes may act synergistically to influence endosymbiont survival under stress conditions (Berrabah et al. 2023).

In addition to age- and stress-induced senescence, ineffective Fix⁻ rhizobium–legume interactions can also trigger premature nodule senescence. Maunoury et al. (2010) classified nonfunctional Fix⁻ nodules from 15 plant and bacterial mutants into 4 distinct categories. Most plant mutants belonged to the first category, including the *M. truncatula* Jemalong 5 mutants TR36, TR183, and TRV36. These mutants formed nodules with elongated, Y-shaped differentiated bacteroids and exhibited 2 waves of nodule-specific transcriptional changes, closely resembling the patterns observed in wild-type nodules. However, early senescence occurred in these nodules as early as 2 weeks after inoculation. While the genetic basis of the TR36 and TRV36 phenotypes remains unknown, the TR183 phenotype has been attributed to the loss of function of the *NODULE-SPECIFIC CYSTEINE-RICH PEPTIDE NEW35 (NCR-new35)* gene (Horváth et al. 2023). Similar nonfunctional nodules were also observed in the *dnf4* (Kim et al. 2015) and *dnf7-2* (Horváth et al. 2015) mutants of *M. truncatula* A17, where deletion of the *NODULE-SPECIFIC CYSTEINE-RICH PEPTIDE 211 (NCR211)* and *NODULE-SPECIFIC CYSTEINE-RICH PEPTIDE 169 (NCR169)* genes, respectively, was responsible for the Fix^−^ phenotype. These NCR genes express consecutively during nodule development, *NCR-new35* in Zone II, *NCR211* in the interzone and in the young nitrogen fixing cells of Zone III while induction of *NCR169* begins in the interzone reaching full expression in Zone III.

The intracellular survival of rhizobia within nodules requires the coordinated activity of plant symbiotic genes, including *DEFECTIVE IN NITROGEN FIXATION 2 (DNF2)*, *Symbiotic CYSTEINE-RICH RECEPTOR-LIKE KINASE (SymCRK*), and *REGULATOR OF SYMBIOSOME DIFFERENTIATION (RSD)*, which encode a phosphatidyl-dependent phospholipase C-like protein, a cysteine-rich receptor kinase, and a C2H2 transcription factor, respectively (Bourcy et al. 2013; Sinharoy et al. 2013; Berrabah et al. 2014). Mutations in these genes result in nonfunctional nodules characterized by heightened defense responses (phenolic accumulation, induction of pathogenesis-related genes) and the death of undifferentiated bacteroids. These insights reveal that the relationship between nodule senescence and immunity in *Medicago* nodules is more complex than previously understood (Berrabah et al. 2023, 2024).

Given these findings, along with numerous examples of premature senescence in *M. truncatula–Sinorhizobium* mutant interactions, we aimed to determine whether this phenomenon is specific to certain mutations or represents a broader feature of unproductive legume–rhizobia symbioses. To investigate this, we analyzed the kinetics of senescence in 5 *M. truncatula* Fix⁻ mutant lines: 3 with characterized defects in *NCR* genes (*dnf4*: *NCR211*, *dnf7-2*: *NCR169*, and TR183: *NCR-new35*), which are activated at consecutive stages of symbiotic cell development in different cell layers, and 2 (TR36 and TRV36) with currently unidentified gene mutations. We found that all 5 mutants exhibited similar senescence-associated traits with some differences in senescence kinetics. These results suggest that early nodule senescence is a common outcome of ineffective *Medicago–Sinorhizobium* symbiotic interactions.

## Results

### Altered nodule growth and *nifH* promoter::*GUS* expression in *dnf4*, *dnf7-2*, TR183, TR36, and TRV36 mutants

**Figure 1.**
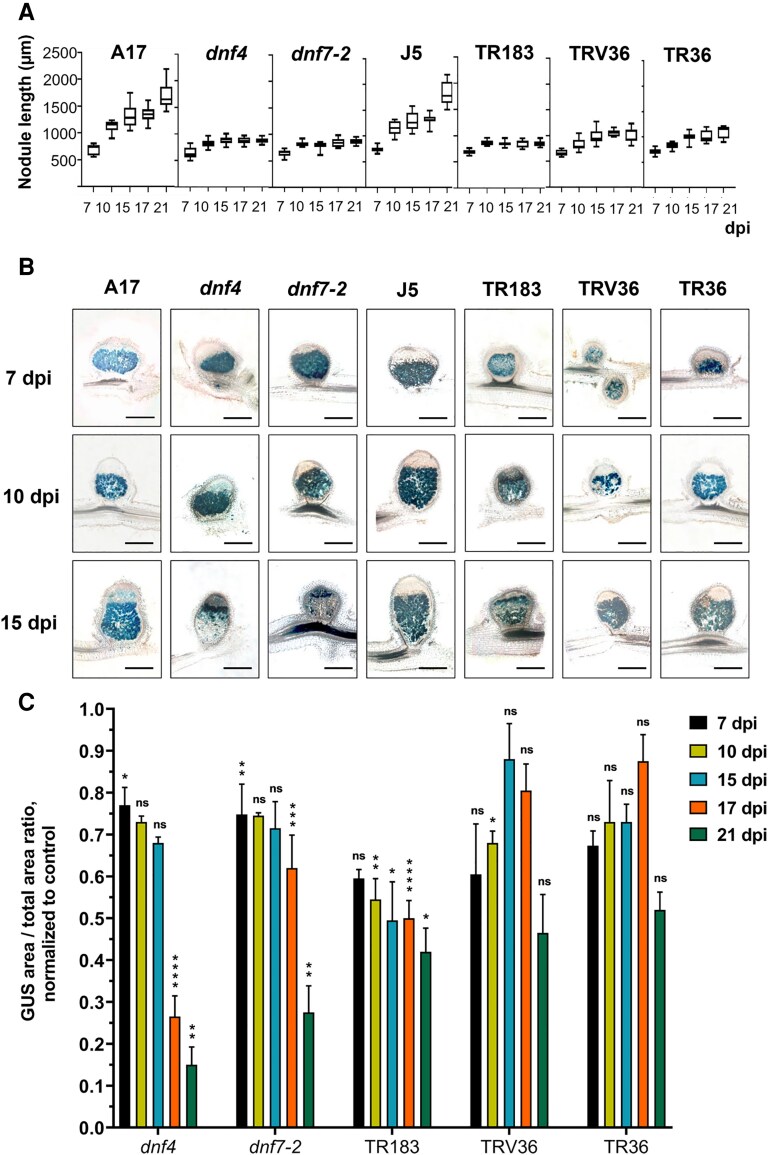
Nodule growth and *PnifH::GUS* expression in wild-type and Fix⁻ nodules. **A)** Nodule size measured at 7, 10, 15, 17, and 21 dpi in wild-type (A17, J5) and Fix⁻ (*dnf4*, *dnf7-2*, TR183, TRV36, TR36) backgrounds. Box plots show the distribution of nodule size at each time point. The center line in the box shows the median, the box limits represent the upper and the lower quartiles, the whiskers show the minimum and the maximum values. Data are from 3 biological replicates each with 7 to 8 nodules from 5 plants per time point. dpi: days postinoculation. **B)** Histochemical GUS staining of agarose-embedded nodule sections at 7, 10, and 15 dpi. Blue coloration indicates *PnifH::GUS* reporter activity. Scale bar: 0.5 mm. **C)** Quantification of plant cells containing bacteria expressing the P*nifH::GUS* reporter gene. Bars represent mean ± SD of GUS-positive area's ratio in nodules normalized to wild-type controls. Asterisks indicate statistically significant differences compared to the respective wild-type control at each time point, as determined by 1-way ANOVA (*P <0.05, **P <0.01, ***P <0.001, ****P <0.0001, ns: not significant). Data are from 3 independent experiments all with 6 to 8 nodules.

The *dnf4* and *dnf7-2* mutants were generated through fast neutron bombardment of *M. truncatula* cv. Jemalong A17 (Starker et al. 2006; Horváth et al. 2015; Kim et al. 2015), while TR183, TRV36, and TR36 resulted from γ-ray mutagenesis of *M. truncatula* cv. Jemalong 5 (Sagan et al. 1995; Morandi et al. 2005; Maunoury et al. 2010). To assess nodule development in these mutants, we compared their growth to that of the respective wild-type ecotypes. The plants were inoculated with *Sinorhizobium medicae* WSM419 carrying the *nifH* promoter::GUS (*PnifH::GUS*) reporter construct (Starker et al. 2006), and nodules were collected at 7, 10, 15, 17, and 21 days postinoculation (dpi). Nodule size, structure, and histochemical GUS staining of the *nifH* promoter::*GUS* reporter on longitudinal sections were observed and measured microscopically from 7 to 21 dpi (Fig. 1, A and B).

The nodule growth (measured by the length of nodules) was similar in the wild-type and mutant plants at 7 dpi. The wild-type A17 and J5 nodules exhibited continuous growth from 7 to 21 dpi, while the nodule size remained unchanged in *dnf4*, *dnf7-2*, and TR183 mutants or exhibited a moderate growth in the case of TRV36 and TR36 nodules (Fig. 1A).

Production of the nitrogenase enzyme was monitored using *nifH* promoter::*GUS* activity (Fig. 1B, Supplementary Fig. S1). At 7 dpi, the GUS staining pattern was comparable between wild-type (A17 and J5) and the *dnf4*, *dnf7-2*, TR183, and TR36 mutant nodules, showing well-defined zonation and strong staining throughout Zone III, indicating proper *nifH* gene expression. Unlike the others, TRV36 displayed fewer GUS-stained symbiotic cells, suggesting a slight developmental delay. At 10 and 15 dpi and later (17 and 21 dpi), the number of GUS-positive cells increased in the wild-type nodules unlike the *dnf4*, *dnf7-2*, and TR183 nodules, where GUS activity was maintained only in the young cells of Zone III. In the TRV36 and TR36 nodules, the number of GUS-stained cells increased during this period but, similar to the other mutants, decreased at the later time points.

To quantify *PnifH::GUS* activity, the GUS-stained blue areas were measured relative to the total nodule area (Fig. 1C). The GUS-positive area significantly decreased in *dnf4* at 17 dpi and in *dnf7-2* at 21 dpi. In TR183, the GUS-stained area was smaller than in the others and gradually decreased, while in TRV36 and TR36, an increase was observed until 15 and 17 dpi, respectively, followed by a decrease in the GUS-positive area.

### Progressive loss of differentiated bacteroids in Fix^−^ mutants of *M. truncatula*

Bacteroid length is a key indicator of their differentiation status. In culture or in an undifferentiated state, *S. medicae* cells typically measure between 1 and 2 μm in length, depending on their cell cycle phase. During symbiosis, however, bacteroids can elongate up to 10 μm, and cells exceeding 2 μm in length are considered to be undergoing differentiation.

**Figure 2.**
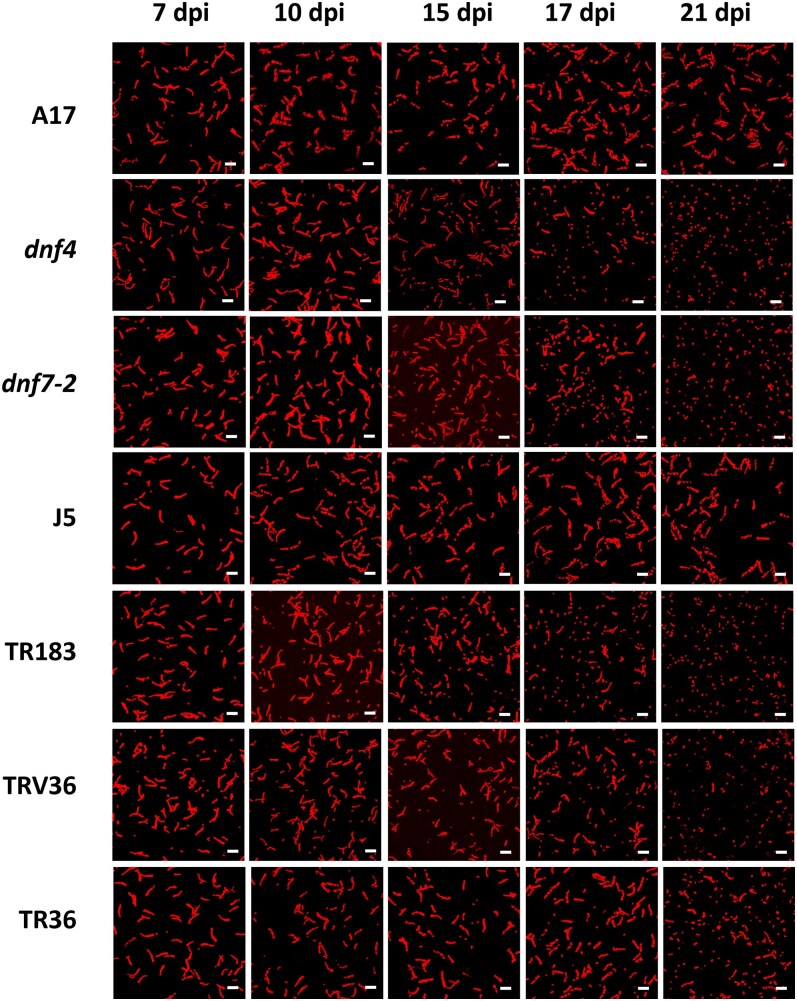
Rhizobial population changes in wild-type and Fix^−^ mutant nodules. Confocal microscopy images of PI-stained bacterial populations isolated from wild-type (A17 and J5) and Fix⁻ mutant (*dnf4*, *dnf7-2*, TR183, TRV36 and TR36) nodules at 7, 10, 15, 17, and 21 dpi. Scale bar: 5 μm. dpi: days postinoculation.

To assess bacteroid differentiation, bacterial populations were isolated from both wild-type and Fix⁻ mutant nodules, boiled, and stained with propidium iodide (PI) for visualization via confocal microscopy (Fig. 2). Elongated bacteroids were already observed in all nodules at 7 dpi. In wild-type nodules, this population of elongated cells was maintained through 21 dpi, the latest time point examined. In contrast, mutant nodules (*dnf4*, *dnf7-2*, TR183, and TRV36) exhibited a progressive decline in elongated bacteroids beginning from 15 or 17 dpi, accompanied by an increasing proportion of small, undifferentiated, saprophytic-like bacteria. By 21 dpi, these undifferentiated forms dominated the bacterial population in all 4 mutants. A similar trend was observed in TR36 nodules, albeit with a slight delay.

**Figure 3.**
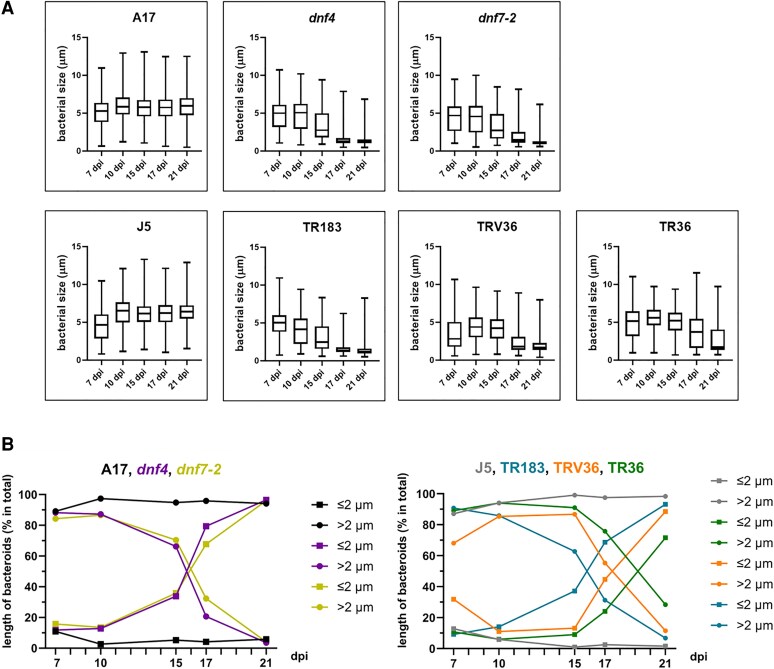
Quantification of bacterial cell size distribution in wild-type and Fix⁻ mutant nodules. **A)** Size distribution of the bacterial populations from 7 to 21 dpi. Measurement of cell lengths was based on confocal images using Olympus FV10-ASW version 4.0 software. The center line in the box shows the median, the box limits represent the upper and the lower quartiles, the whiskers show the minimum and the maximum values. dpi: days postinoculation. **B)** Kinetics of the formation of differentiated bacteroids (>2 μm) and the appearance of saprophytic bacteria (≤2 μm). Data are from 3 biological replicates (*n* = 500 to 1,000 cells).

To quantify these observations, bacteroid cell lengths were determined from confocal images, measuring at each time point 500 to 1,000 cells (Fig. 3, Supplementary Table S1). At 7 dpi, bacteroids longer than 2 μm comprised 87.1% to 89.1% of the total population in wild-type A17 and J5 nodules, indicating robust early differentiation. Most mutants also showed normal bacteroid development at this stage, with the exception of TRV36, where differentiation was delayed, as 31.9% of the isolated cells remained small and undifferentiated.

Analysis of size distribution over time (Figs. 2 and 3A) revealed a slight increase in bacteroid length in wild-type nodules from 7 to 10 dpi, followed by a stable plateau. In *dnf4* and *dnf7-2* nodules, bacteroids at 7 and 10 dpi were also elongated and comparable in size to the wild type. However, the average lengths of the bacterial population decreased at later time points, with *dnf4* exhibiting a faster decline than *dnf7-2*. In TR183, the length of rhizobial population decreased steadily over time. In TRV36 and TR36, a slight increase in bacteroid size was observed initially, but this was followed by a marked reduction from 17 dpi onward. Overall, in all Fix⁻ mutant nodules, elongated bacteroids gradually declined and were eventually replaced by small, saprophytic-like rhizobia, resembling the morphology of cultured cells—indicating a breakdown in symbiotic differentiation.

Although all Fix⁻ mutants followed this general pattern, the timing and extent of senescence varied, as shown by the proportion of ≤2 μm versus >2 μm bacteroids (Fig. 3B). In wild-type nodules, this proportion remained stable from 10 dpi onward. In contrast, *dnf4*, *dnf7-2*, and TR183 mutants showed a rapid decline in >2 μm bacteroids and a corresponding increase in smaller cells starting from 15 dpi. TRV36 and TR36 displayed a similar shift, but with a delayed onset at 17 dpi. Notably, TR36 retained a small proportion (28.4%) of elongated bacteroids even at 21 dpi, while the other mutants were nearly fully populated by small, undifferentiated bacteria at this stage (Supplementary Table S1).

### Rapidly progressing nodule senescence is accompanied by closed endodermis

Longitudinal growth of *M. truncatula* nodules is maintained by a persistent apical nodule meristem, which continuously generates postmitotic cells that enter the differentiation process and give rise to various nodule cell types. In Fix⁻ mutants, nodule size was generally smaller compared to wild-type plants. To examine structural differences between wild-type and mutant nodules in greater detail, nodules induced with *S. medicae* WSM419 were harvested at 7 and 17 dpi, embedded in agarose, and sectioned at 75-μm thickness. Sections were stained with Syto 9 (green fluorescence) to detect live bacteroids, while the nodule endodermis was visualized by its blue autofluorescence under UV light (Tirichine et al. 2000).

**Figure 4.**
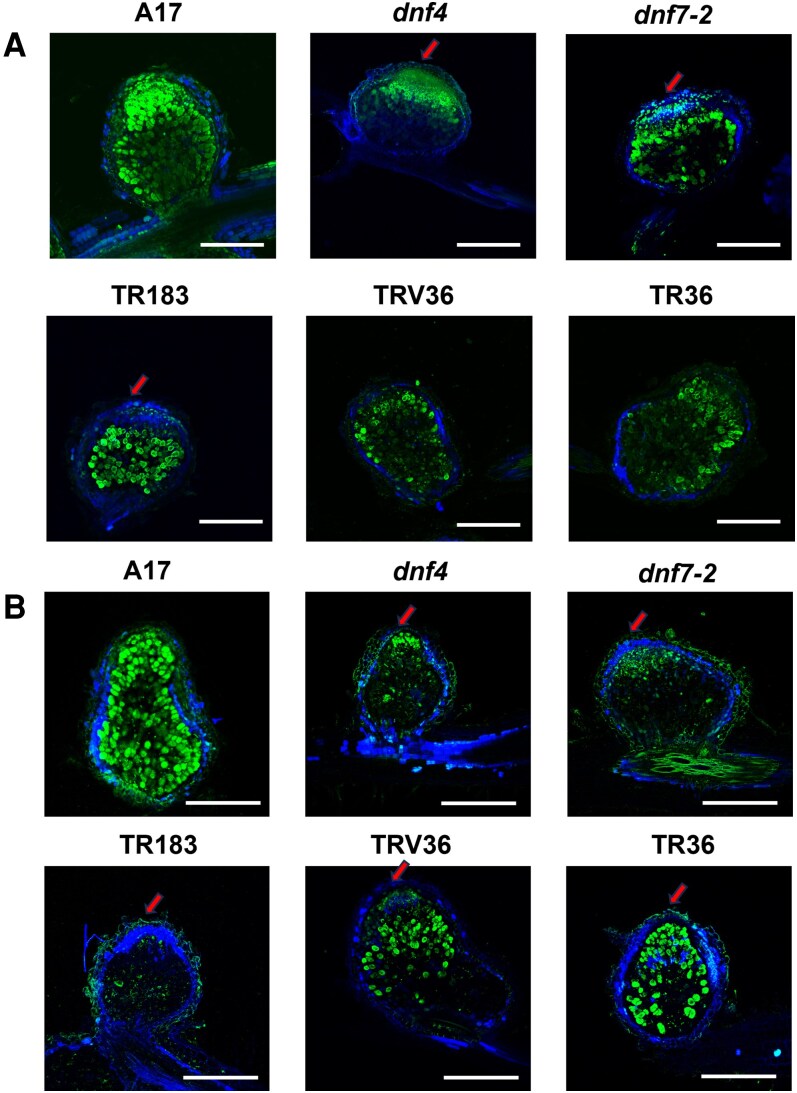
Early endodermis closure in ineffective nodules. Visualization of endodermis and symbiotic cells with live bacteroids in longitudinal sections of wild-type (A17) and mutant (*dnf4*, *dnf7-2*, TR183, TRV36, and TR36) *M. truncatula* nodules at 7 dpi **(A)** and 17 dpi **(B)**. Endodermis shows blue autofluorescence, while live bacteria stained with Syto 9 are green. Red arrows indicate the closed endodermis at the nodule apex. Scale bar: 250 μm **(A)**, 500 μm **(B)**. dpi: days postinoculation.

At 7 dpi, all nodules contained symbiotic cells with live bacteroids, although these were more abundant in the wild-type nodules. Notably, even at this early stage, the endodermis was already closed above the meristem in the *dnf4*, *dnf7-2*, and TR183 nodules, whereas it remained open in wild-type (A17 and J5) nodules and in TRV36 and TR36 nodules (Fig. 4A). At 17 dpi, in contrast to the wild type, the endodermis was closed in all mutant nodules. Furthermore, large symbiotic cells were absent in *dnf4*, *dnf7-2*, and TR183 nodules, while TRV36 and TR36 nodules more closely resembled wild-type nodules at this stage (Fig. 4B).

**Figure 5.**
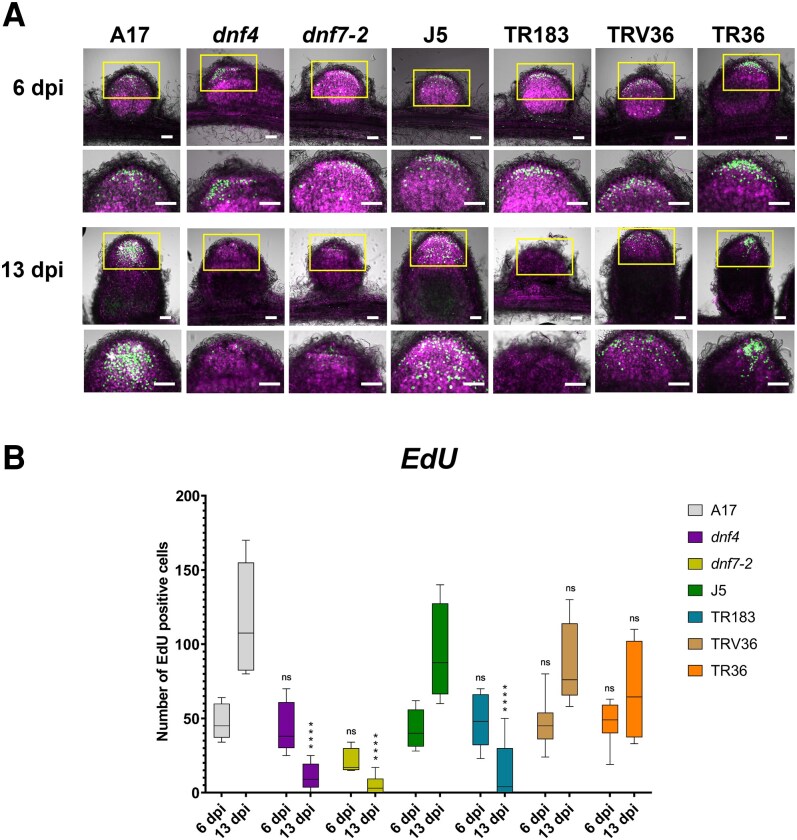
Detection and quantification of de novo synthesized DNA in wild-type and Fix^−^ nodules at 6 and 13 dpi. **A)** In wild-type and mutant nodules, nuclei undergoing de novo DNA synthesis are marked by EdU incorporation (green fluorescence), while nuclei not labeled with EdU were stained with DAPI and pseudocolored magenta in confocal images. Yellow rectangles indicate enlarged regions shown below each image. Scale bar: 100 μm. Dpi: days postinoculation. **B)** Quantification of EdU-positive cells in wild-type and mutant nodules. Box plots show the number of EdU-positive cells per mutants. The center line in the box shows the median, the box limits represent the upper and the lower quartiles, the whiskers show the minimum and the maximum values. Data are from 3 independent experiments (*n* = 6 to 8 nodules). Asterisks indicate statistically significant differences compared to the respective wild-type control at each time point, as determined by 1-way ANOVA (*P < 0.05, **P < 0.01, ***P < 0.001, ****P < 0.0001, ns: not significant).

To assess the cell cycle activity in the wild-type and mutant nodules, de novo DNA synthesis was monitored at 6 and 13 dpi nodules with incorporation of EdU (5-ethynyl-2′-deoxyuridine) for 15 h. Then reaction of Alexa-488-labeled azide with the incorporated EdU resulted in bright green fluorescence of de novo synthetized DNA in the S-phase nuclei of the mitotic cell cycle or nuclei undergoing DNA synthesis during endoreduplication cycles (Vanstraelen et al. 2009). After removing the excess of fluorophore-labeled azide, the nodules were counterstained with DAPI to also visualize all nonlabeled nuclei, which were pseudocolored in magenta on the confocal images (Fig. 5A). At 6 dpi, in the early stage of development, EdU-labeled nuclei were present in all nodules, mostly in the meristem but also in nuclei undergoing endoreduplication in Zone II. At 13 dpi, EdU fluorescence was hardly detectable in the *dnf4*, *dnf7-2*, and TR183 nodules, indicating the complete decline of cell cycle activity and disappearance of meristem. At this stage, the meristem was still functional and endoreduplication of the genome occurred in Zone II in the TRV36, TR36, and wild-type nodules as indicated by EdU fluorescence.

To quantitatively assess nuclei undergoing de novo DNA synthesis, the total number of EdU-positive cells was counted in wild-type and mutant nodules at 6 and 13 dpi (Fig. 5B). At 6 dpi, the number of EdU-positive cells was similar across all genotypes, except for TR183, which showed a reduced number of labeled cells. By 13 dpi, a marked increase in EdU-positive cells was observed in wild-type nodules, indicating active DNA synthesis. A moderate increase was also detected in TRV36 and TR36 nodules. In contrast, *dnf4*, *dnf7-2*, and TR183 mutants exhibited a sharp decline in EdU-positive cell numbers, suggesting a premature cessation of DNA synthesis and impaired cell proliferation and/or endoreduplication in these nodules.

### Reduced proportion of high-ploidy nuclei in Fix^−^ mutant nodules

**Figure 6.**
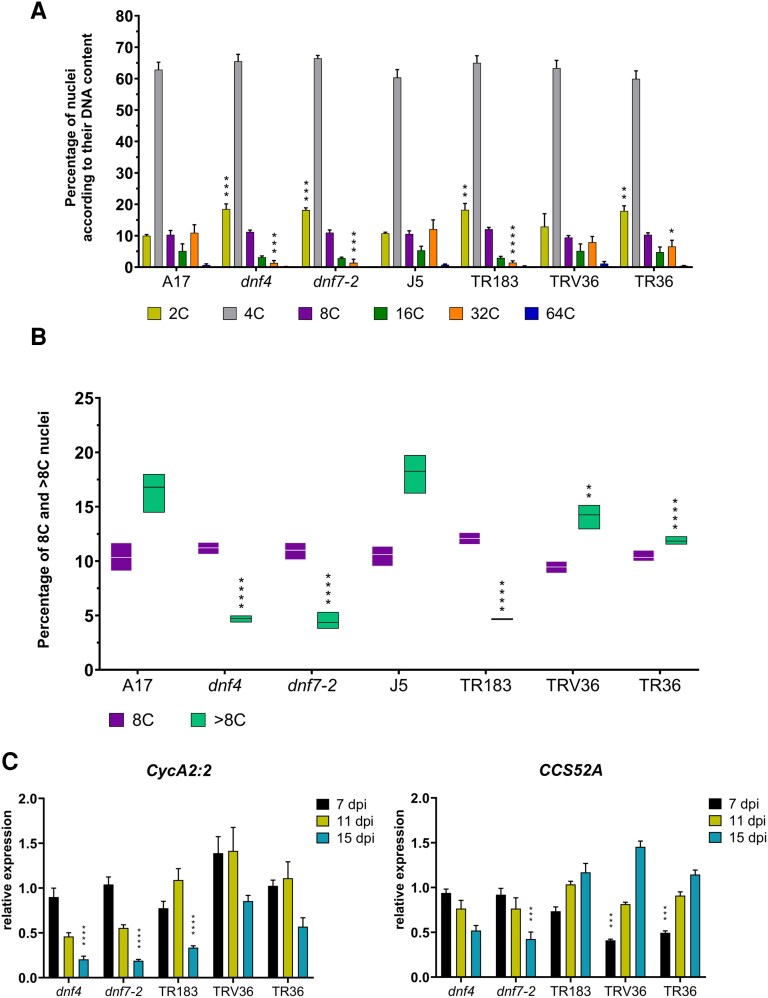
Flow cytometric analysis of nuclear ploidy and expression of cell cycle marker genes. **A)** DNA content of nuclei in wild-type *M. truncatula* A17 and J5 and Fix⁻ mutant (*dnf4*, *dnf7-2*, TR183, TRV36, and TR36) nodules at 15 dpi. Data are from 3 biological replicates. dpi: days postinoculation. **B)** Box plot presentation of the percentage of 8C and >8C nuclei in each genotype. The center line in the box shows the median, the box limits represent the upper and the lower quartiles, and the whiskers show the minimum and the maximum values. **C)** Relative expression of the cell cycle-related genes *CCS52A* and *CycA2:2* in nodules at 7, 11, and 15 dpi, measured by RT-qPCR expression values normalized to wild-type levels represent means ± SD from 3 biological replicates with 6 to 8 nodules. Asterisks indicate statistical significance (*P < 0.05, **P < 0.01, ***P < 0.001, ****P < 0.0001), as determined by 1-way ANOVA. Bars without asterisk showed no significant difference.

The observed decline in EdU incorporation in Fix⁻ mutant nodules suggests a reduction in both meristematic activity and endoreduplication in symbiotic cells. To investigate the extent of polyploidization, we analyzed the DNA content of nuclei isolated from wild-type and mutant nodules at 15 dpi. Nuclei were stained with PI and sorted according to their DNA content (1C corresponds to the haploid genome) into 2C, 4C, 8C, 16C, 32C, and 64C populations using flow cytometry (Fig. 6A).

As established previously, nodule cells exit the meristem with 4C DNA content (Yang et al. 1994; Cebolla et al. 1999); thus, 8C content indicates completion of the first round of endoreduplication. Across all samples, 4C nuclei constituted the predominant population, and the abundance of 8C nuclei was comparable among wild-type and mutant nodules (Fig. 6, A and B). However, in *dnf4*, *dnf7-2*, TR183, and TR36 nodules, the proportion of 2C nuclei was elevated, while the percentages of 32C and 64C nuclei were reduced. This suggests that although the initial round of endoreduplication occurs in these mutants, subsequent rounds—particularly the third and fourth—are markedly impaired. Interestingly, the ploidy distribution in TRV36 and TR36 nodules more closely resembled that of the wild-type J5 nodules, likely due to the later onset or slower progression of senescence in these lines.

While 8C nuclei were present at similar levels across all genotypes, the total percentage of nuclei with >8C DNA content varied considerably. In wild-type A17 and J5 nodules, high-ploidy (>8C) nuclei accounted for 16.2% and 18.1% of the total population, respectively. In contrast, this proportion dropped sharply in the *dnf4*, *dnf7-2*, and TR183 mutants (4.5% to 5.0%) and was moderately reduced in TRV36 (14.0%) and TR36 (11.8%) (Fig. 6B, Supplementary Table S2).

To get an insight into the cell cycle activity of the wild-type and mutant nodules, RT-qPCR experiments were performed to measure transcript levels of the A2 type *CYCLIN (CycA2:2)*, a marker for cell proliferation (Roudier et al. 2003), and *CELL CYCLE SWITCH GENE 52A (CCS52A),* required for cell differentiation and the induction of endoreduplication cycles (Cebolla et al. 1999; Vinardell et al. 2003). In 15-dpi nodules, *CycA2:2* expression was downregulated in all mutant nodules, with a progressive decrease in transcript levels observed in *dnf4* and *dnf7-2* from 7 to 15 dpi (Fig. 6C). The *CCS52A* expression (Fig. 6C) followed a similar trend in these mutants, with declining transcript levels over time, consistent with impaired differentiation. In TR183, TRV36, and TR36 nodules, *CCS52A* expression was lower at 7 dpi compared to wild-type J5 nodules. However, in TRV36 and TR36, transcript levels increased at 11 and 15 dpi, reflecting their developmental delay. In TR183, *CCS52A* expression reached wild-type levels by 11 and 15 dpi, yet the symbiotic cells still failed to develop highly polyploid nuclei, indicating that restored *CCS52A* expression alone was insufficient to drive full endoreduplication and terminal differentiation.

### Leghemoglobin expression is impaired in Fix^−^ mutants

Leghemoglobin plays a crucial role in supporting biological nitrogen fixation by protecting the oxygen-sensitive nitrogenase enzyme in rhizobia. Structurally, the nodule's cortical cell layers and endodermis create an effective oxygen diffusion barrier, while leghemoglobin (Lb) buffers intracellular oxygen concentrations within infected plant cells. This dual mechanism ensures a balance between respiration and nitrogen fixation (Larrainzar et al. 2020). The heme moiety of Lb, synthesized by the plant (Wang et al. 2025), gives nodules their characteristic pink coloration, serving as a visual indicator of active nitrogen fixation. During nodule senescence, Lb is degraded by proteases (Pladys and Vance 1993), and the released heme is catabolized by heme oxygenases into ferrous iron and biliverdin. The latter contributes to the green coloration typically observed in senescing nodules (Zhou et al. 2023).

**Figure 7.**
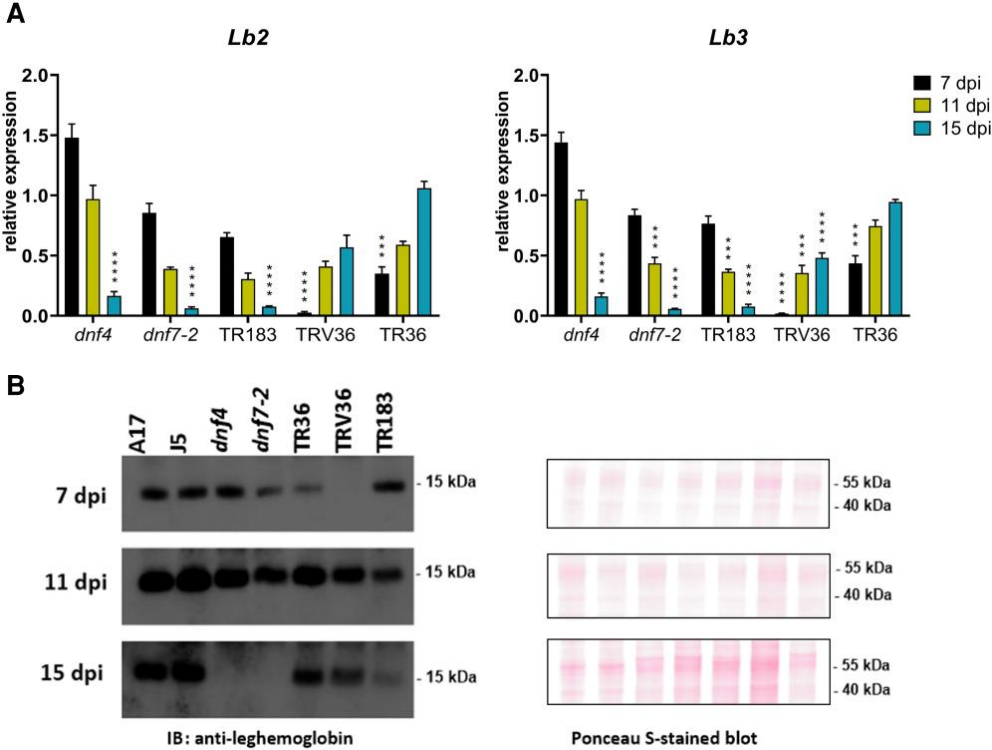
Dynamics of Lb mRNA and protein levels in wild-type and *dnf4*, *dnf7-2*, TR183, TRV36, and TR36 Fix^−^ mutant nodules. **A)** Relative expression levels of the *Lb2* and *Lb3* genes by RT-qPCR analysis at 7, 11, and 15 dpi. Expression values (from 3 independent experiments) are normalized to wild types, and only the mutants are presented. Asterisks indicate statistical significance (*P < 0.05, **P < 0.01, ***P < 0.001, ****P < 0.0001), as determined by 1-way ANOVA. dpi: days postinoculation. **B)** Western immunoblot analysis of Lb protein levels with anti-Lb antibodies (left panel) and Ponceau-S-stained blot as a loading control (right panel).

The premature closure of the endodermis observed in Fix⁻ mutants led us to hypothesize that Lb production or stability may be compromised in these genotypes. To test this, we analyzed the mRNA levels of 2 Lb genes, *LEGHEMOGLOBIN 2* (*Lb2 Medtr1g011540*; Gallusci et al. 1991; Xi et al. 2013) and *LEGHEMOGLOBIN 3* (*Lb*3; *Medtr5g081000*; Cabeza et al. 2014), using RT-qPCR in wild-type and Fix⁻ mutant nodules (Fig. 7A). Expression of both *Lb2* and *Lb3* significantly declined by 15 dpi in *dnf4*, *dnf7-2*, and TR183 mutants, which also exhibited early-onset nodule senescence. In contrast, TRV36 and TR36 mutants—characterized by delayed nodule development—showed low or barely detectable *Lb2* and *Lb3* expression at 7 dpi. However, expression levels progressively increased over time, reaching near wild-type levels in TR36 and moderately reduced levels in TRV36 by 15 dpi, in alignment with their developmental progression.

Given that *M. truncatula* possesses 12 Lb genes, we also assessed overall Lb protein levels in wild-type and Fix⁻ mutant nodules using western blot analysis with anti-Lb antibodies (Fig. 7B). Consistent with the gene expression data, relatively high Lb protein levels were detected at 7 dpi in wild-type A17 and J5 nodules, as well as in the *dnf4*, *dnf7-2*, and TR183 mutants. In contrast, TR36 showed lower levels, while TRV36 exhibited no detectable Lb protein at this stage. By 11 dpi, Lb protein levels increased further in both wild-type and mutant nodules, with the exception of TR183, where levels began to decline. At 15 dpi, Lb protein remained abundant in A17, J5, TR36, and TRV36 nodules, whereas it dropped to undetectable levels in *dnf4* and *dnf7-2* and was markedly reduced in TR183. These protein dynamics align well with the observed *Lb2* and *Lb3* transcript patterns, supporting a correlation between impaired Lb accumulation and early nodule senescence in the Fix⁻ mutants.

### Early induction of senescence marker genes without defense gene activation in the Fix^−^ mutant nodules

Under normal conditions, nodules undergo developmental senescence approximately 4 weeks after inoculation. However, in *dnf4*, *dnf7-2*, and TR183 mutants, signs of bacteroid degradation and arrested nodule development were observed as early as 10 dpi, while in TR36 and TRV36, these processes began at 15 dpi. Proteases play a central role in nodule senescence by breaking down symbiotic cells and recycling their components to support biosynthetic processes in other parts of the plant. Among these, cysteine proteases (CPs) serve as early molecular markers of nodule senescence (Van de Velde et al. 2006; Pérez Guerra et al. 2010; Cam et al. 2012).

**Figure 8.**
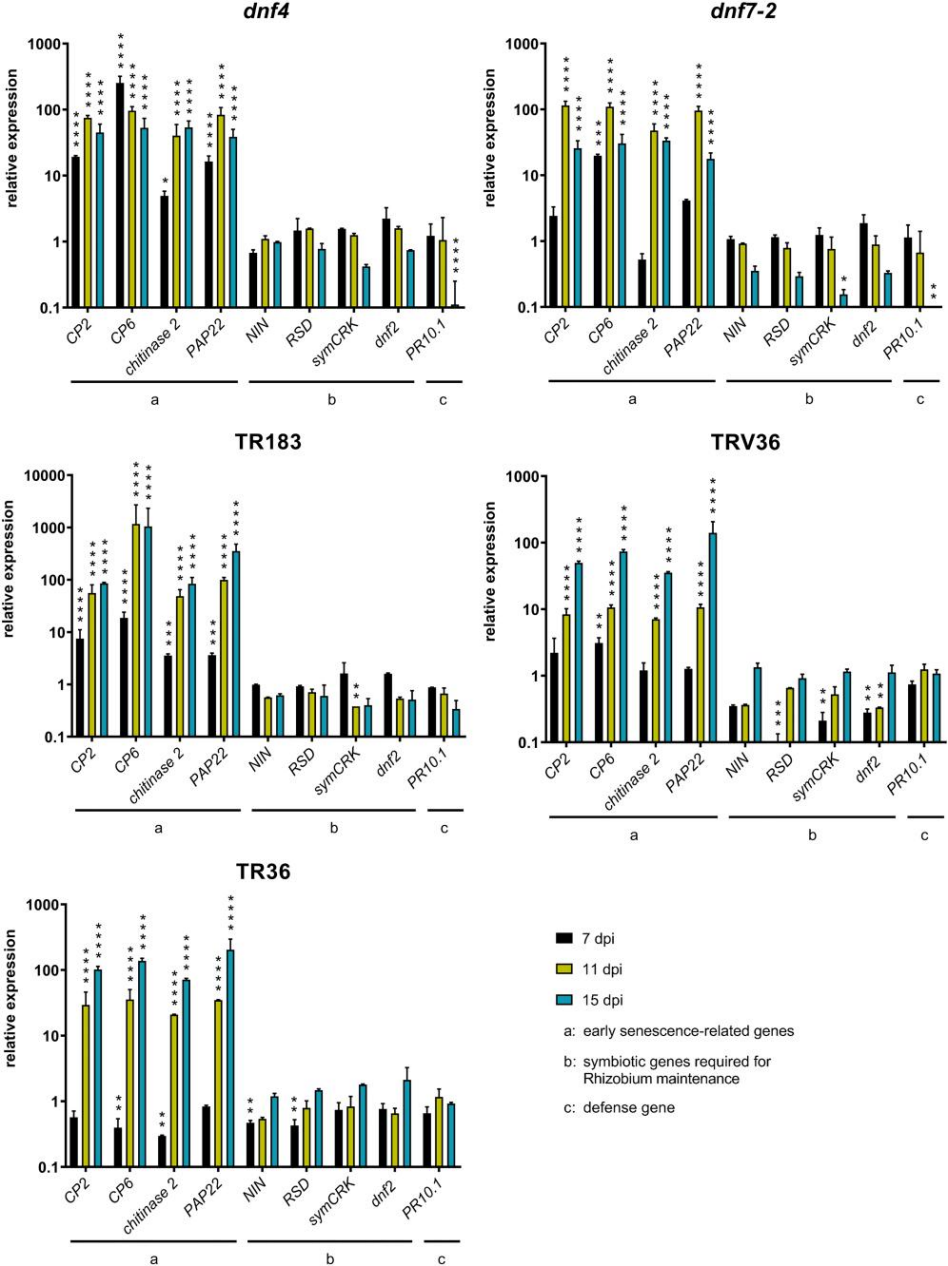
RT-qPCR analysis of marker genes involved in senescence, symbiotic immunity, and plant defense in wild-type and Fix^−^ mutant nodules at 7, 11, and 15 dpi. Relative expression levels in the mutants were normalized to the respective wild-type lines (data are plotted on a logarithmic scale). Note that in the TR183 panel, the *y*-axis scale is 1 order of magnitude higher than in the other graphs. Bars represent mean ± SD from 3 independent experiments. Asterisks indicate statistically significant differences compared to the respective wild-type control at each time point, as determined by 1-way ANOVA (*P < 0.05, **P < 0.01, ***P < 0.001, ****P < 0.0001). Bars without significance markers showed no significant difference. dpi: days postinoculation.

To assess the onset of senescence at 7, 11, and 15 dpi in both wild-type and Fix⁻ mutant nodules at the molecular level, we quantified the transcript levels of genes in 3 categories using RT-qPCR that were (a) senescence-related genes: *CYSTEINE PROTEASE 2* (*CP2*), *CYSTEINE PROTEASE 6* (*CP6*), *CHITINASE 2*, and *PURPLE ACID PHOSPHATASE 22* (*PAP22*); (b) symbiotic genes: *NODULE INCEPTION* (*NIN*; Liu et al. 2021), *RSD*, *SymCRK*, and *DNF2*; and (c) the defense-related *PATHOGENESIS-RELATED Protein 10.1* (*PR10.1*) gene (for references and gene IDs, see Supplementary Table S3) in wild-type and mutant nodules at 7, 11, and 15 dpi using RT-qPCR (Fig. 8).

The senescence marker genes were upregulated in all mutants by 11 dpi and in the rapidly senescing *dnf4*, *dnf7-2*, and TR183 mutants even by 7 dpi, compared to the wild-type A17 and J5 controls except *CHITINASE 2* in *dnf7-2* mutant whose expression level increased only from 11 dpi (Fig. 8). In TR183, expression of *CP6* increased 1,000-fold at 11 and 15 dpi compared to J5 and was higher than in the other early senescing mutants.

Unlike senescence marker genes, the expression of symbiotic genes involved in maintaining bacteroid survival, suppression of defense, and premature senescence was not upregulated in any of the mutants. Instead, transcript levels of some of these genes were lower in *dnf4*, *dnf7-2*, and TR183 nodules compared to wild-type controls, suggesting that reduced expression of these genes may contribute to premature bacteroid death.

The expression pattern of *PR10.1* mirrored that of the symbiotic genes. Transcript levels of *PR10.1* in all mutants were comparable to the wild-type at 7 and 11 dpi, while at 15 dpi, even a decrease in *PR10.1* expression was observed in the early senescing *dnf4*, *dnf7-2*, and TR183 mutants, while TR36 and TRV36 maintained stable expression levels.

These findings indicate that early senescence in these mutant nodules is primarily driven by the premature upregulation of senescence-related genes and thus increased proteolytic activity, rather than the activation of host defense mechanisms.

## Discussion

Our study provides compelling evidence that premature nodule senescence is a common outcome in Fix⁻ *M. truncatula* mutants, despite differences in their underlying genetic defects. The rapid decline in the expression of nitrogen-fixation-related genes, early degradation of bacteroids, and the upregulation of senescence marker genes observed across all mutants suggest that ineffective symbiotic interactions universally trigger nodule deterioration. The absence of upregulation in symbiotic maintenance genes such as *NIN*, *DNF2*, *SymCRK*, and *RSD* and the defense-related gene *PR10.1* further supports the hypothesis that early nodule senescence in these mutants is primarily driven by proteolytic degradation rather than an immune response.

A critical aspect of our findings is the comparison between natural nodule senescence and early senescence in Fix⁻ mutants. Natural senescence is a highly regulated and progressive process that occurs usually after 30 dpi as a function of nodule aging, allowing for the controlled degradation and recycling of cellular components without abrupt disruption of nitrogen fixation. It is driven by developmental cues, hormonal regulation, and metabolic changes that gradually lead to a decline in nodule function (Van de Velde et al. 2006). In contrast, early senescence in Fix⁻ mutants is characterized by an accelerated loss of symbiotic capacity, often associated with structural defects and rapid bacteroid degradation. While both processes involve increased proteolytic activity (Pierre et al. 2014), early senescence is distinct in that it lacks the carefully orchestrated transitions seen in natural senescence. Instead, it is often triggered prematurely by genetic deficiencies that impair bacteroid differentiation and thus prevents the onset of nitrogen fixation and results in an abrupt cessation of further nodule development.

One key difference between natural and early senescence is the regulation of defense-related genes. During natural senescence, defense genes may be upregulated at later stages as part of a coordinated degradation process. This includes the activation of pathogenesis-related (PR) genes, proteases, and oxidative stress regulators that contribute to the breakdown of symbiotic cells (Berrabah et al. 2023, 2024). In contrast, early senescence in Fix⁻ mutants does not involve a robust activation of defense-related genes. Our findings indicate that *PR10.1* expression remains stable or even declines in Fix⁻ nodules, suggesting that early senescence is not accompanied by a strong immune response. This is in contrast to defense-associated nodule senescence triggered by environmental stress, where significant activation of PR genes is observed. The lack of defense gene upregulation in Fix⁻ mutants implies that premature senescence is primarily a consequence of metabolic imbalance and failed symbiotic differentiation rather than an active immune-driven degradation of nodules.

The structural and physiological alterations in Fix⁻ nodules, particularly the premature closure of the nodule endodermis, appear to play a critical role in accelerating senescence. Endodermal closure was detected as early as 7 dpi in *dnf4*, *dnf7-2*, and TR183 nodules, and at 17 dpi in all Fix⁻ mutants, preceding or coinciding with meristem exhaustion and bacteroid collapses. The presence of a suberized endodermal layer surrounding the nodule apex may have different consequences: it can form a rigid or sealed boundary and physically restrict further cell division in the meristem and restrict the uptake of water and ions while the lignified and suberized endodermis encasing the vascular bundles may hinder nutrient exchange and thus further nodule development (Tirichine et al. 2000). Moreover, the endodermis and the surrounding parenchymal cortical cell layers function as O_2_ diffusion barriers that modulates oxygen availability, contributing an optimal balance between respiration and nitrogen fixation (Minchin 1997). In Fix⁻ mutants, premature closure of the endodermis likely restricts water and ion uptake as well as oxygen diffusion. Combined with reduced Lb transcript and protein levels, these factors may lead to a decline in ATP production and metabolic activity, ultimately impairing symbiotic cell differentiation and bacteroid function.

The dynamics of nodule growth, bacteroid differentiation and cell cycle progression in Fix⁻ mutants provide additional insights into the relationship between meristem maintenance and premature senescence. Our EdU labeling experiments revealed that while meristematic activity was initially comparable to wild-type nodules, it rapidly declined in Fix⁻ mutants exhibiting early senescence. This was supported by a marked drop in the number of EdU-positive nuclei at 13 dpi, and reduced frequency of >8C ploidy nuclei in flow cytometry analyses. This correlates with a significant reduction in the expression of *CycA2:2* and *CCS52A*, markers of cell cycle activity and endoreduplication, respectively. The reduced number of high-ploidy nuclei in these mutants further supports the idea that compromised cell cycle regulation contributes to the arrest of symbiotic cell differentiation, leading to an early transition into senescence. The progressive decline of meristematic activity, followed by the differentiation of meristematic cells into infected nodule cells containing terminally differentiated bacteroids, leads to the loss of young, proliferating bacteroids in Zone II. It can be speculated that this population of immature bacteroids, potentially through the production of Nod factors, phytohormones or other molecules, may play a role in maintaining meristem activity during the functional lifespan of the nodule.

Interestingly, while all Fix⁻ mutants shared a general trend of premature senescence, the timing of bacteroid degradation and loss of meristematic activity varied. In the *dnf4, dnf7-2*, and TR183 mutants these processes began as early as 10 dpi, whereas in the TR36 and TRV36 mutants, which exhibited somewhat delayed nodule development, they started after 15 dpi, with relatively preserved meristematic activity and a more gradual decline in Lb levels. This suggests that the different mutations permit symbiotic cell differentiation to varying degrees and for different durations before eventual senescence ensues. The underlying molecular mechanisms governing these differences remain unclear but may involve variations in the extent of oxygen limitation, metabolic stress, or signals between developing bacteroids and the host cell. Missing signals from bacteroids could also impact the host's ability to receive and provide the necessary cues for subsequent developmental steps, full bacteroid differentiation, and nodule longevity.

Our findings reinforce the idea that early nodule senescence in Fix⁻ mutants is a highly coordinated process, integrating oxygen regulation, proteolytic activity, and meristematic maintenance. Unlike developmental senescence, which occurs as a regulated aging process, premature senescence in Fix⁻ mutants appears to be a consequence of impaired symbiotic differentiation, metabolic imbalances, and the failure to sustain bacteroid viability. Understanding these mechanisms is crucial for improving the efficiency of legume-rhizobium symbiosis, particularly in agricultural contexts where enhanced nodule longevity could improve nitrogen fixation and plant productivity.

Future research should focus on elucidating the specific molecular triggers that initiate premature senescence in Fix⁻ mutants. Comparative transcriptomic and proteomic analyses could provide further insights into the differential regulatory pathways that distinguish early senescing mutants from those with delayed nodule deterioration. Additionally, genetic modifications aimed at modulating endodermis permeability, oxygen buffering capacity, or protease activity could help identify potential strategies to extend nodule function in ineffective symbiotic interactions. These approaches will contribute to a broader understanding of nodule senescence and may inform biotechnological efforts to optimize nitrogen-fixing symbioses for sustainable agriculture.

## Materials and methods

### Plant material, growth conditions, and inoculation

Barrel medic (*M. truncatula* cv. Jemalong A17 and Jemalong 5) plants were used as wild-type controls when the *dnf4* (Starker et al. 2006; Kim et al. 2015), *dnf7-2* (Starker et al. 2006; Horváth et al. 2015), TR36 (Sagan et al. 1995; Morandi et al. 2005), TRV36 (Maunoury et al. 2010), and TR183 (Sagan et al. 1995; Morandi et al. 2005) Fix^−^ mutants were studied. Plants were grown as described in the *M. truncatula* Handbook (https://www.noble.org/medicago-handbook/). For EdU labeling, *in vitro*-grown plants were cultured on vertical plates containing Gibson buffered nodulation medium supplemented with 1 mM aminoethoxyvinylglycine (AVG; 15546, Cayman Chemical) (Ehrhardt et al. 1992). Plants were inoculated with *S. medicae* WSM419 harboring the *PnifH::GUS* reporter gene construct (Starker et al. 2006).

### Microscopic analyses

Seven to 8 nodules from 4 to 5 plants per time point per biological replicates were collected at indicated time points. To determine the size distribution of the isolated bacterial population, the nodules were frozen in liquid nitrogen, ground to a fine powder, and resuspended in 1× PBS (pH 7.4), and the nodule debris was removed by brief centrifugation (100 × *g*, 5 min). Cleared supernatant was filtered with CellTrics (10 μm mesh, Sysmex Partec). The bacteroids were pelleted by centrifugation (4400 × *g*, 10 min). For confocal microscopy, the boiled bacteroids were stained with PI for 5 min at a final concentration of 30 µM. Live/dead staining of bacteria was performed on fresh nodule sections in PBS (pH 7.4) containing 5 µM Syto 9 and 30 µM PI (LIVE/DEAD BacLight Viability Kit, Life Technologies) for 20 min at RT. Sections were rinsed with ultrapure water and examined with an Olympus FluoView FV1000 (Olympus) or Leica SP5 (Leica) confocal laser scanning microscope. The blue autofluorescence of the endodermis (under UV light) was also recorded. The length of the isolated bacteroids (10 images per line and time point (from 3 independent experiments), ∼500 to 1,000 cells) was measured with the microscope's dedicated software (Olympus FV10-ASW version 4.0) by measuring the length of a straight line connecting the 2 endpoints of a cell along the main axis. Histochemical GUS staining was performed on formaldehyde-fixed nodules (Horváth et al. 2015), and images were captured with a Zeiss Axioskop 40 (Zeiss) microscope. To determine GUS reporter activity, the area of blue-stained tissue was measured in ImageJ using the freehand selection tool (Schindelin et al. 2012). The GUS-positive area and total nodule area were measured separately, and relative signal intensity was expressed as the GUS-stained area divided by total nodule area. Values were normalized to the corresponding wild-type controls at the same time point (dpi).

### EdU labeling

Root system of plants was completely submerged in 10 mL of liquid Gibson medium containing 30 μM EdU (5-ethynyl-2′-deoxyuridine) in 50 mL standard polypropylene conical tubes. After 15 h of EdU labeling, the samples were fixed with freshly prepared 4% paraformaldehyde containing 0.1% Triton X-100 in PBS for 1 h. Alexa 488 azide-based EdU detection and sample preparation for confocal microscopy were performed (Vanstraelen et al. 2009; Kotogány et al. 2010). Briefly, nodules and 0.5 cm-long root tips were cut into 96-well plates containing PBS. Root tips were also included in the experiment as a positive control for EdU labeling and detection. PBS was replaced in each well with freshly prepared EdU detection cocktail (prepared according to the manufacturer's instructions) and shaken at 200 rpm in the dark for 1 h. The cocktail was then replaced with 1 μg/mL of DAPI (4′,6-diamidino-2-phenylindole) in PBS for 1 h. Samples were washed once with PBS for 15 min and mounted with Fluoromount-G antifade solution (Southern Biotechnology Assoc., Birmingham, AL, USA). The number of EdU-positive nuclei was determined manually using the multipoint tool in ImageJ software (Schindelin et al. 2012). Maximum-intensity projections of confocal images were analyzed, and each EdU-labeled nucleus was marked by sequential clicking, which automatically generated a total count displayed on screen.

### Gene expression analysis

Twenty to 30 nodules from 6 to 8 plants per line per time point and biological replicates collected at 7, 11, and 15 dpi were immediately frozen in liquid nitrogen. Total RNA was isolated with the ZR Plant RNA Miniprep kit (Zymo Research). For cDNA synthesis, 100 ng of total RNA was reverse-transcribed in a 20 μL of reaction using the High-Capacity cDNA Reverse Transcription Kit (Applied Biosystems). RT-qPCR was performed with the StepOne Plus (Thermo Fisher Scientific) using the PowerUp SYBR Green Master Mix (Applied Biosystems) according to the manufacturer's instructions. Cycle thresholds were determined, and data were analyzed using the StepOne software v2.3. Relative expression levels were calculated by the ΔΔCt method using the 40S ribosomal protein S19 gene as reference gene (Nagymihály et al. 2017). The primers used in this study are listed in Supplementary Table S3.

### Flow cytometry

Nodules were chopped in nuclei isolation buffer consisting of 45 mM MgCl_2_, 20 mM MOPS, 30 mM sodium citrate, and 0.1% Triton X-100 (pH 7.0) (Galbraith et al. 1983). Nuclei released from the cells were filtered through 30 μm nylon sieves and stained with PI (10 μM final concentration) and then analyzed with Beckman MoFlo Astrios (Beckman Coulter) flow cytometer.

### Immunoblotting

Total soluble protein extract was prepared from nodules collected at indicated time points in a buffer containing 50 mM Tris-HCl (pH 7.4), 50 mM NaCl, 1 mM EDTA, 0.5% Triton X-100, and protease inhibitor cocktail (Complete, Merck Millipore). The protein concentration of cleared samples was determined using the Pierce Detergent Compatible Bradford Assay Kit (Thermo Fisher Scientific). Protein samples were separated in 12% SDS–polyacrylamide gels, blotted onto PVDF membrane (Immobilon-P, Merck Millipore) and immunoprobed with anti-Lb polyclonal antibody. Ponceau S-staining served as loading control. Anti-Lb (*M. sativa* Lb3), a generous gift from Dr Carroll P Vance (University of Minnesota USDA ARS, Wycoff 1998; Cordoba et al. 2003), was diluted 1:1,000. As secondary antibody goat anti-rabbit IgG (H + L)-HRP Conjugate #1706515 (Bio-Rad Laboratories) was used.

### Accession numbers

Sequence data from this article can be found in the GenBank/EMBL data libraries under accession numbers Medtr7g029760: NCR169; Medtr4g035705: NCR211; Medtr0512s0030: NCR-new35; Medtr2g102520: CycA2:2; Medtr4g102510: CCS52A; Medtr1g011540: Lb2; Medtr5g081000: Lb3; Medtr5g022560: CP2; Medtr4g079800: CP6; Medtr6g079630: CHITINASE; Medtr7g104360: PAP22; Medtr5g099060: NIN; Medtr7g063220: RSD; Medtr3g079850: SymCRK; Medtr4g085800: DNF2; Medtr4g120970: PR10.1; Medtr3g013635: 40S.

## Supplementary Material

kiaf518_Supplementary_Data

## Data Availability

The data underlying this article are included within the article itself and in the accompanying online supplementary materials.

## Supplementary data

The following materials are available in the online version of this article.

**Supplementary Figure S1.** GUS staining of *PnifH::GUS* reporter activity in wild-type and Fix⁻ mutant nodules at 17 and 21 dpi. Scale bar: 0.5 mm

**Supplementary Table S1.** Quantification of bacteroid differentiation arrest in Fix^−^ nodules

**Supplementary Table S2.** Quantification of the DNA content of nuclei isolated from nodules

**Supplementary Table S3.** Primers used in this study

## Dive Curated Terms

The following phenotypic, genotypic, and functional terms are of significance to the work described in this paper:

PAP22 Gramene: Medtr7g104360
PAP22 Araport: Medtr7g104360

NCR169 Gramene: Medtr7g029760
NCR169 Araport: Medtr7g029760

NCR211 Gramene: Medtr4g035705
NCR211 Araport: Medtr4g035705

NCR-new35 Gramene: Medtr0512s0030
NCR-new35 Araport: Medtr0512s0030

CycA2:2 Gramene: Medtr2g102520
CycA2:2 Araport: Medtr2g102520

CP2 Gramene: Medtr5g022560
CP2 Araport: Medtr5g022560

CP6 Gramene: Medtr4g079800
CP6 Araport: Medtr4g079800

CCS52A Gramene: Medtr4g102510
CCS52A Araport: Medtr4g102510

CHITINASE Gramene: Medtr6g079630
CHITINASE Araport: Medtr6g079630

NIN Gramene: Medtr5g099060
NIN Araport: Medtr5g099060

RSD Gramene: Medtr7g063220
RSD Araport: Medtr7g063220

SymCRK Gramene: Medtr3g079850
SymCRK Araport: Medtr3g079850

DNF2 Gramene: Medtr4g085800
DNF2 Araport: Medtr4g085800

PR10.1 Gramene: Medtr4g120970
PR10.1 Araport: Medtr4g120970

40S Gramene: Medtr3g013635
40S Araport: Medtr3g013635

Lb2 Gramene: Medtr1g011540
Lb2 Araport: Medtr1g011540

Lb3 Gramene: Medtr5g081000
Lb3 Araport: Medtr5g081000

## References

[kiaf518-B1] Berrabah F, Benaceur F, Yin C, Xin D, Magne K, Garmier M, Gruber V, Ratet P. Defense and senescence interplay in legume nodules. Plant Commun. 2024:5(4):100888. 10.1016/j.xplc.2024.100888 38532645 PMC11009364

[kiaf518-B2] Berrabah F, Bernal G, Elhosseyn A-S, El Kassis C, L’Horset R, Benaceur F, Wen J, Mysore KS, Garmier M, Gourion B, et al Insight into the control of nodule immunity and senescence during *Medicago truncatula* symbiosis. Plant Physiol. 2023:191(1):729–746. 10.1093/plphys/kiac505 36305683 PMC9806560

[kiaf518-B3] Berrabah F, Bourcy M, Cayrel A, Eschstruth A, Mondy S, Ratet P, Gourion B. Growth conditions determine the DNF2 requirement for symbiosis. PLoS One. 2014:9(3):e91866. 10.1371/journal.pone.0091866 24632747 PMC3954807

[kiaf518-B4] Bourcy M, Brocard L, Pislariu CI, Cosson V, Mergaert P, Tadege M, Mysore KS, Udvardi MK, Gourion B, Ratet P. *Medicago truncatula* DNF2 is a PI-PLC-XD-containing protein required for bacteroid persistence and prevention of nodule early senescence and defense-like reactions. New Phytol. 2013:197(4):1250–1261. 10.1111/nph.12091 23278348

[kiaf518-B5] Cabeza R, Koester B, Liese R, Lingner A, Baumgarten V, Dirks J, Salinas-Riester G, Pommerenke C, Dittert K, Schulze J. An RNA sequencing transcriptome analysis reveals novel insights into molecular aspects of the nitrate impact on the nodule activity of *Medicago truncatula*. Plant Physiol. 2014:164(1):400–411. 10.1104/pp.113.228312 24285852 PMC3875817

[kiaf518-B6] Cam Y, Pierre O, Boncompagni E, Hérouart D, Meilhoc E, Bruand C. Nitric oxide (NO): a key player in the senescence of *Medicago truncatula* root nodules. New Phytol. 2012:196(2):548–560. 10.1111/j.1469-8137.2012.04282.x 22937888

[kiaf518-B7] Cebolla A, Vinardell JM, Kiss E, Oláh B, Roudier F, Kondorosi A, Kondorosi E. The mitotic inhibitor ccs52 is required for endoreduplication and ploidy-dependent cell enlargement in plants. EMBO J. 1999:18(16):4476–4484. 10.1093/emboj/18.16.4476 10449413 PMC1171522

[kiaf518-B8] Cordoba E, Shishkova S, Vance CP, Hernández G. Antisense inhibition of NADH glutamate synthase impairs carbon/nitrogen assimilation in nodules of alfalfa (*Medicago sativa* L.). Plant J. 2003:33(6):1037–1049. 10.1046/j.1365-313X.2003.01686.x 12631328

[kiaf518-B9] Ehrhardt DW, Atkinson EM, Long SR. Depolarization of alfalfa root hair membrane potential by *Rhizobium meliloti* nod factors. Science. 1992:256(5059):998–1000. 10.1126/science.10744524 10744524

[kiaf518-B10] Escuredo PR, Minchin FR, Gogorcena Y, Iturbe-Ormaetxe I, Klucas RV, Becana M. Involvement of activated oxygen in nitrate-induced senescence of pea root nodules. Plant Physiol. 1996:110(4):1187–1195. 10.1104/pp.110.4.1187 12226252 PMC160906

[kiaf518-B11] Galbraith DW, Harkins KR, Maddox JM, Ayres NM, Sharma DP, Firoozabady E. Rapid flow cytometric analysis of the cell cycle in intact plant tissues. Science. 1983:220(4601):1049–1051. 10.1126/science.220.4601.1049 17754551

[kiaf518-B12] Gallusci P, Dedieu A, Journet EP, Huguet T, Barker DG. Synchronous expression of leghaemoglobin genes in *Medicago truncatula* during nitrogen-fixing root nodule development and response to exogenously supplied nitrate. Plant Mol Biol. 1991:17(3):335–349. 10.1007/BF00040629 1883994

[kiaf518-B13] Geurts R, Bisseling T. *Rhizobium* nod factor perception and signalling. Plant Cell. 2002:14(suppl 1):s239–s249. 10.1105/tpc.002451 12045280 PMC151258

[kiaf518-B14] Gogorcena Y, Gordon AJ, Escuredo PR, Minchin FR, Witty JF, Moran JF, Becana M. N2 fixation, carbon metabolism, and oxidative damage in nodules of dark-stressed common bean plants. Plant Physiol. 1997:113(4):1193–1201. 10.1104/pp.113.4.1193 12223669 PMC158242

[kiaf518-B15] Gogorcena Y, Iturbe-Ormaetxe I, Escuredo PR, Becana M. Antioxidant defenses against activated oxygen in pea nodules subjected to water stress. Plant Physiol. 1995:108(2):753–759. 10.1104/pp.108.2.753 12228507 PMC157397

[kiaf518-B16] González EM, Aparicio-Tejo PM, Gordon AJ, Minchin FR, Royuela M, Arrese-Igor C. Water-deficit effects on carbon and nitrogen metabolism of pea nodules. J Exp Bot. 1998:49(327):1705–1714. 10.1093/jxb/49.327.1705

[kiaf518-B17] Horváth B, Domonkos Á, Kereszt A, Szűcs A, Ábrahám E, Ayaydin F, Bóka K, Chen Y, Chen R, Murray JD, et al Loss of the nodule-specific cysteine rich peptide, NCR169, abolishes symbiotic nitrogen fixation in the *Medicago truncatula dnf7* mutant. Proc Natl Acad Sci U S A. 2015:112(49):15232–15237. 10.1073/pnas.1500777112 26401023 PMC4679056

[kiaf518-B18] Horváth B, Güngör B, Tóth M, Domonkos Á, Ayaydin F, Saifi F, Chen Y, Biró JB, Bourge M, Szabó Z, et al The *Medicago truncatula* nodule-specific cysteine-rich peptides, NCR343 and NCR-new35 are required for the maintenance of rhizobia in nitrogen-fixing nodules. New Phytol. 2023:239(5):1974–1988. 10.1111/nph.19097 37381081

[kiaf518-B19] Kim M, Chen Y, Xi J, Waters C, Chen R, Wang D. An antimicrobial peptide essential for bacterial survival in the nitrogen-fixing symbiosis. Proc Natl Acad Sci U S A. 2015:112(49):15238–15243. 10.1073/pnas.1500123112 26598690 PMC4679048

[kiaf518-B20] Kondorosi E, Mergaert P, Kereszt A. A paradigm for endosymbiotic life: cell differentiation of *rhizobium* bacteria provoked by host plant factors. Annu Rev Microbiol. 2013:67(1):611–628. 10.1146/annurev-micro-092412-155630 24024639

[kiaf518-B21] Kotogány E, Dudits D, Horváth GV, Ayaydin F. A rapid and robust assay for detection of S-phase cell cycle progression in plant cells and tissues by using ethynyl deoxyuridine. Plant Methods. 2010:6(1):5. 10.1186/1746-4811-6-5 20181034 PMC2828981

[kiaf518-B22] Larrainzar E, Villar I, Rubio MC, Pérez-Rontomé C, Huertas R, Sato S, Mun J-H, Becana M. Hemoglobins in the legume–*Rhizobium* symbiosis. New Phytol. 2020:228(2):472–484. 10.1111/nph.16673 32442331

[kiaf518-B23] Liu J, Rasing M, Zeng T, Klein J, Kulikova O, Bisseling T. NIN is essential for development of symbiosomes, suppression of defence and premature senescence in *Medicago truncatula* nodules. New Phytol. 2021:230(1):290–303. 10.1111/nph.17215 33471433 PMC7986424

[kiaf518-B24] Lorenzo C, Lucas MM, Vivo A, de Felipe MR. Effect of nitrate on peroxisome ultrastructure and catalase activity in nodules of *Lupinus albus* L. cv. Multolupa. J Exp Bot. 1990:41(12):1573–1578. 10.1093/jxb/41.12.1573

[kiaf518-B25] Matamoros MA, Baird LM, Escuredo PR, Dalton DA, Minchin FR, Iturbe-Ormaetxe I, Rubio MC, Moran JF, Gordon AJ, Becana M. Stress-induced legume root nodule senescence. Physiological, biochemical, and structural alterations. Plant Physiol. 1999:121(1):97–112. 10.1104/pp.121.1.97 10482665 PMC59394

[kiaf518-B26] Maunoury N, Redondo-Nieto M, Bourcy M, Van de Velde W, Alunni B, Laporte P, Durand P, Agier N, Marisa L, Vaubert D, et al Differentiation of symbiotic cells and endosymbionts in *Medicago truncatula* nodulation are coupled to two transcriptome-switches. PLoS One. 2010:5(3):e9519. 10.1371/journal.pone.0009519 20209049 PMC2832008

[kiaf518-B27] Mergaert P, Uchiumi T, Alunni B, Evanno G, Cheron A, Catrice O, Mausset A-E, Barloy-Hubler F, Galibert F, Kondorosi A, et al Eukaryotic control on bacterial cell cycle and differentiation in the *Rhizobium*–legume symbiosis. Proc Natl Acad Sci U S A. 2006:103(13):5230–5235. 10.1073/pnas.0600912103 16547129 PMC1458823

[kiaf518-B28] Minchin FR . Regulation of oxygen diffusion in legume nodules. *Soil Biol Biochem*. 1997:29(5–6):881–888. 10.1016/S0038-07179600204-0

[kiaf518-B29] Morandi D, Prado E, Sagan M, Duc G. Characterisation of new symbiotic *Medicago truncatula* (Gaertn.) mutants, and phenotypic or genotypic complementary information on previously described mutants. Mycorrhiza. 2005:15(4):283–289. 10.1007/s00572-004-0331-4 15558330

[kiaf518-B30] Nagymihály M, Veluchamy A, Györgypál Z, Ariel F, Jégu T, Benhamed M, Szűcs A, Kereszt A, Mergaert P, Kondorosi É. Ploidy-dependent changes in the epigenome of symbiotic cells correlate with specific patterns of gene expression. Proc Natl Acad Sci U S A. 2017:114(17):4543–4548. 10.1073/pnas.1704211114 28404731 PMC5410778

[kiaf518-B31] Oldroyd GED, Murray JD, Poole PS, Downie JA. The rules of engagement in the legume–rhizobial symbiosis. Annu Rev Genet. 2011:45(1):119–144. 10.1146/annurev-genet-110410-132549 21838550

[kiaf518-B32] Pérez Guerra JC, Coussens G, De Keyser A, De Rycke R, De Bodt S, Van De Velde W, Goormachtig S, Holsters M. Comparison of developmental and stress-induced nodule senescence in *Medicago truncatula*. Plant Physiol. 2010:152(3):1574–1584. 10.1104/pp.109.151399 20081044 PMC2832273

[kiaf518-B33] Pierre O, Hopkins J, Combier M, Baldacci F, Engler G, Brouquisse R, Hérouart D, Boncompagni E. Involvement of papain and legumain proteinase in the senescence process of *Medicago truncatula* nodules. New Phytol. 2014:202(3):849–863. 10.1111/nph.12717 24527680

[kiaf518-B34] Pladys D, Vance CP. Proteolysis during development and senescence of effective and plant gene-controlled ineffective alfalfa nodules. Plant Physiol. 1993:103(2):379–384. 10.1104/pp.103.2.379 12231944 PMC158993

[kiaf518-B35] Roudier F, Fedorova E, Lebris M, Lecomte P, Györgyey J, Vaubert D, Horvath G, Abad P, Kondorosi A, Kondorosi E. The *Medicago* species A2-type cyclin is auxin regulated and involved in meristem formation but dispensable for endoreduplication-associated developmental programs. Plant Physiol. 2003:131(3):1091–1103. 10.1104/pp.102.011122 12644661 PMC166874

[kiaf518-B36] Sagan M, Morandi D, Tarenghi E, Duc G. Selection of nodulation and mycorrhizal mutants in the model plant *Medicago truncatula* (Gaertn.) after γ-ray mutagenesis. Plant Sci. 1995:111(1):63–71. 10.1016/0168-9452(95)04229-N

[kiaf518-B37] Schindelin J, Arganda-Carreras I, Frise E, Kaynig V, Longair M, Pietzsch T, Preibisch S, Rueden C, Saalfeld S, Schmid B, et al Fiji: an open-source platform for biological-image analysis. Nat Methods. 2012:9(7):676–682. 10.1038/nmeth.2019 22743772 PMC3855844

[kiaf518-B38] Seabra AR, Pereira PA, Becker JD, Carvalho HG. Inhibition of glutamine synthetase by phosphinothricin leads to transcriptome reprograming in root nodules of *Medicago truncatula*. Mol Plant Microbe Interact. 2012:25(7):976–992. 10.1094/MPMI-12-11-0322 22414438

[kiaf518-B39] Sinharoy S, Torres-Jerez I, Bandyopadhyay K, Kereszt A, Pislariu CI, Nakashima J, Benedito VA, Kondorosi E, Udvardi MK. The C_2_H_2_ transcription factor REGULATOR OF SYMBIOSOME DIFFERENTIATION represses transcription of the secretory pathway gene *VAMP721a* and promotes symbiosome development in *Medicago truncatula*. Plant Cell. 2013:25(9):3584–3601. 10.1105/tpc.113.114017 24082011 PMC3809551

[kiaf518-B40] Starker CG, Parra-Colmenares AL, Smith L, Mitra RM, Long SR. Nitrogen fixation mutants of *Medicago truncatula* fail to support plant and bacterial symbiotic gene expression. Plant Physiol. 2006:140(2):671–680. 10.1104/pp.105.072132 16407449 PMC1361333

[kiaf518-B41] Swaraj K, Laura JS, Bishnoi NR. Nitrate induced nodule senescence and changes in activities of enzymes scavenging H_2_O_2_ in clusterbean (*Cyamopsis tetragonaloba* Taub.). J Plant Physiol. 1993:141(2):202–205. 10.1016/S0176-1617(11)80760-1

[kiaf518-B42] Tirichine L, de Billy F, Huguet T. *Mtsym6*, a gene conditioning *Sinorhizobium* strain-specific nitrogen fixation in *Medicago truncatula*. Plant Physiol. 2000:123(3):845–852. 10.1104/pp.123.3.845 10889234 PMC59048

[kiaf518-B43] Van de Velde W, Guerra JCP, Keyser AD, Rycke RD, Rombauts S, Maunoury N, Mergaert P, Kondorosi E, Holsters M, Goormachtig S. Aging in legume symbiosis. A molecular view on nodule senescence in *Medicago truncatula*. Plant Physiol. 2006:141(2):711–720. 10.1104/pp.106.078691 16648219 PMC1475454

[kiaf518-B44] Vanstraelen M, Baloban M, Da Ines O, Cultrone A, Lammens T, Boudolf V, Brown S, C D, Veylder L, Mergaert P, et al APC/C^CCS52A^ complexes control meristem maintenance in the *Arabidopsis* root. Proc Natl Acad Sci U S A. 2009:106(28):11806–11811. 10.1073/pnas.0901193106 19553203 PMC2710644

[kiaf518-B45] Vasse J, de Billy F, Camut S, Truchet G. Correlation between ultrastructural differentiation of bacteroids and nitrogen fixation in alfalfa nodules. J Bacteriol. 1990:172(8):4295–4306. 10.1128/jb.172.8.4295-4306.1990 2376562 PMC213254

[kiaf518-B46] Vinardell JM, Fedorova E, Cebolla A, Kevei Z, Horvath G, Kelemen Z, Tarayre S, Roudier F, Mergaert P, Kondorosi A, et al Endoreduplication mediated by the anaphase-promoting complex activator CCS52A is required for symbiotic cell differentiation in *Medicago truncatula* nodules. Plant Cell. 2003:15(9):2093–2105. 10.1105/tpc.014373 12953113 PMC181333

[kiaf518-B47] Wang L, Tian T, Deng Y, Ji J, Liang J, Guan Y, Li R, Huang X, Wang Y, Ning G, et al Plant glutamyl-tRNA reductases coordinate plant and rhizobial heme biosynthesis in nitrogen-fixing nodules. Plant Cell. 2025:37(5):koaf095. 10.1093/plcell/koaf095 40315358 PMC12107067

[kiaf518-B48] Wycoff K . Effects of oxygen on nodule physiology and expression of nodulins in alfalfa. Plant Physogy. 1998:117(2):385–395. 10.1104/pp.117.2.385 PMC34958 9625691

[kiaf518-B49] Xi J, Chen Y, Nakashima J, Wang S-m, Chen R. *Medicago truncatula esn1* defines a genetic locus involved in nodule senescence and symbiotic nitrogen fixation. Mol Plant Microbe Interact. 2013:26(8):893–902. 10.1094/MPMI-02-13-0043-R 23634841

[kiaf518-B50] Yang WC, de Blank C, Meskiene I, Hirt H, Bakker J, van Kammen A, Franssen H, Bisseling T. Rhizobium nod factors reactivate the cell cycle during infection and nodule primordium formation, but the cycle is only completed in primordium formation. Plant Cell. 1994:6(10):1415–1426. 10.1105/tpc.6.10.1415 7994175 PMC160530

[kiaf518-B51] Zhou Y, Wang L, Rubio MC, Pérez-Rontomé C, Zhou Y, Qi Y, Tian T, Zhang W, Fan Q, Becana M, et al Heme catabolism mediated by heme oxygenase in uninfected interstitial cells enables efficient symbiotic nitrogen fixation in *Lotus japonicus* nodules. New Phytol. 2023:239(5):1989–2006. 10.1111/nph.19074 37329247

