# Supplementary figures and images for: Diverse triggers, common outcome: Senescence in Fix⁻ Medicago truncatula nodules
Source: Plant Physiol. 2025 Oct 23;199(3):kiaf518. doi: 10.1093/plphys/kiaf518 (PMC12610396; doi:10.1093/plphys/kiaf518)

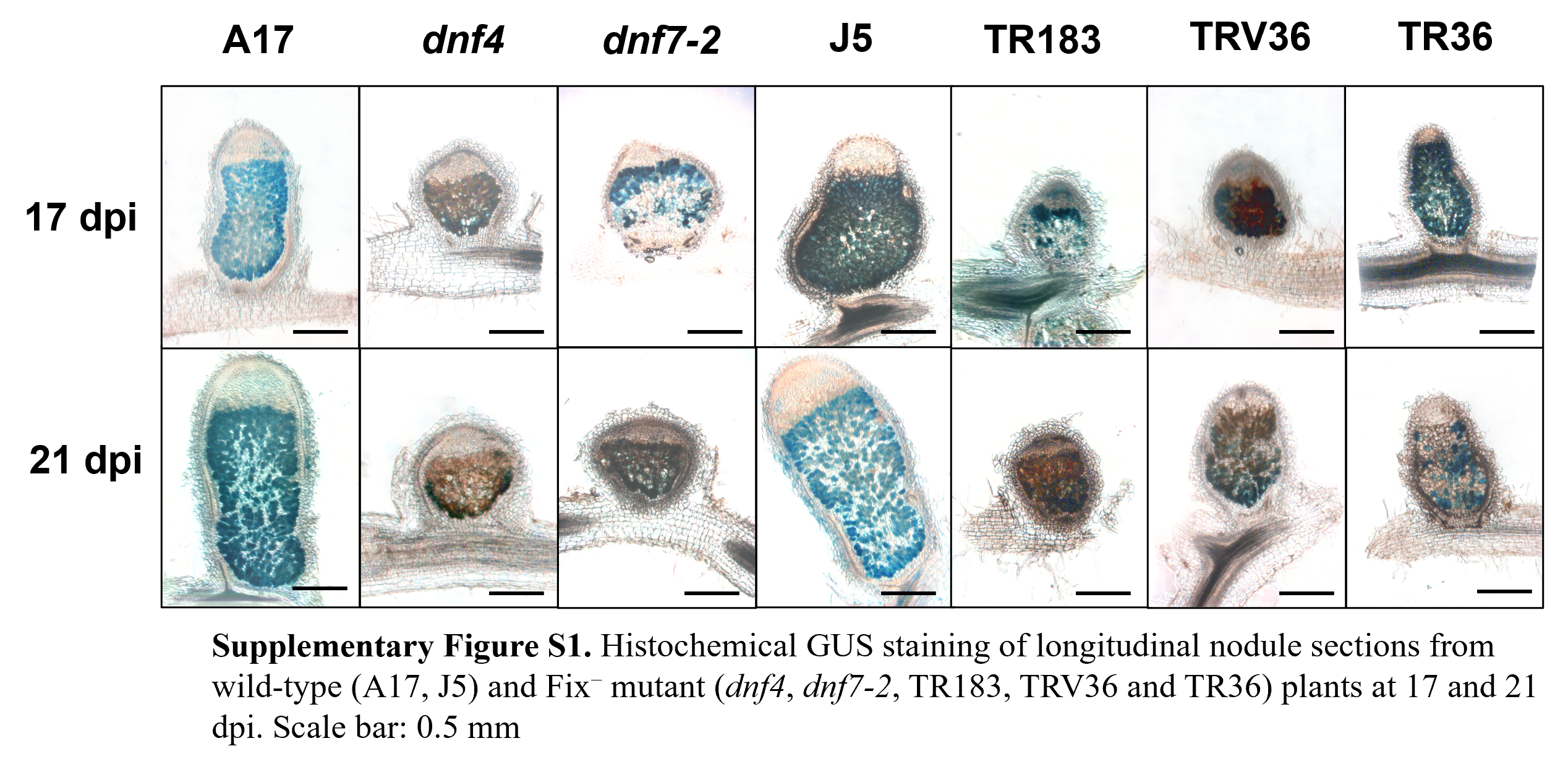

Supplement: kiaf518_Supplementary_Data [file kiaf518_supplementary_data.zip › Supplementary Data.tif]
